# Vacuum exhausted isolation locker (VEIL) to reduce inpatient droplet/aerosol transmission during COVID-19 pandemic

**DOI:** 10.1017/ice.2020.1414

**Published:** 2021-01-11

**Authors:** Tilvawala Gopesh, Alex M. Grant, Jessica H. Wen, Teresa H. Wen, Ernesto Criado-Hidalgo, William J. Connacher, James R. Friend, Timothy A. Morris

**Affiliations:** 1 Medically Advanced Devices Laboratory, Center for Medical Devices Department of Mechanical and Aerospace Engineering, Jacobs School of Engineering, University of California – San Diego, La Jolla, California; 2 Prototyping Laboratory, Qualcomm Institute, University of California – San Diego, La Jolla, California; 3 Division of Pulmonary, Critical Care and Sleep Medicine, University of California – San Diego, San Diego, California

## Abstract

The vacuum-exhausted isolation locker (VEIL) provides a safety barrier during the care of COVID-19 patients. The VEIL is a 175-L enclosure with exhaust ports to continuously extract air through viral particle filters connected to hospital suction. Our experiments show that the VEIL contains and exhausts exhaled aerosols and droplets.

Hospitals continue to care for large numbers of coronavirus disease 2019 (COVID-19) patients, many of whom have hypoxemic respiratory compromise^1^ and are at high risk of life-threatening respiratory failure. Early treatment with noninvasive ventilation therapies that utilize high air flows, such as nasal high flow oxygen (NHF) and BiPAP, provide benefit to similar patients with hypoxemic respiratory compromise, which decrease progression to respiratory failure, need for mechanical ventilation, and mortality.^2,3^ However, clinicians have been reluctant to use these therapies in COVID-19 patients,^4^ possibly due to the perceived risk of viral transmission to hospital staff in close proximity to patients undergoing potentially aerosol-generating procedures.^5,6^ The high gas-flow rates inherent to those therapies are thought to more easily spread droplets to the surrounding environment.^5^ A recent report described a physical barrier enclosure that prevented transmission of cough-induced particles onto the mask and gown of a laryngoscopist during the brief amount of time required for endotracheal intubation.^7^ Here, we report on a newly developed vacuum-exhausted isolation locker (VEIL) that effectively contains and exhausts patient-exhaled droplets and aerosols round-the-clock while patients receive advanced respiratory therapies.

## Methods

**Fig. 1.**
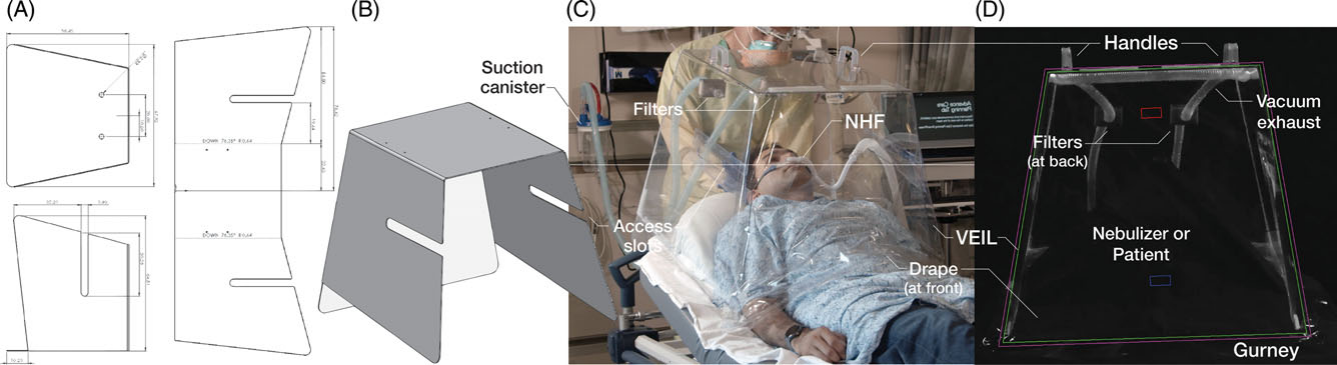
Polycarbonate sheet (A) is thermobent to form the vacuum-exhausted isolation locker (VEIL) shell (B). The subject lays supine inside the shell which is then enclosed by a polyvinylchloride drape (C). A darkfield (fluorescent) view from the foot of the gurney (D) facilitates imaging of aerosols and droplets in and outside the VEIL.

The VEIL is a 175-L polycarbonate enclosure formed via thermal bending and closed at the inferior end with a clear polyvinylchloride drape (Fig. 1). The VEIL is placed at the head of a bed or gurney over the patient’s torso and can be removed easily for emergencies (http://veil.ucsd.edu). Noninvasive ventilation tubing, including the larger corrugated tubing utilized by NHF, is passed via flap-closed horizontal slots in the enclosure, minimizing air leakage. Exhaust ports continuously extract air from the VEIL through viral particle filters that are connected to the standard hospital suction system.

**Fig. 2.**
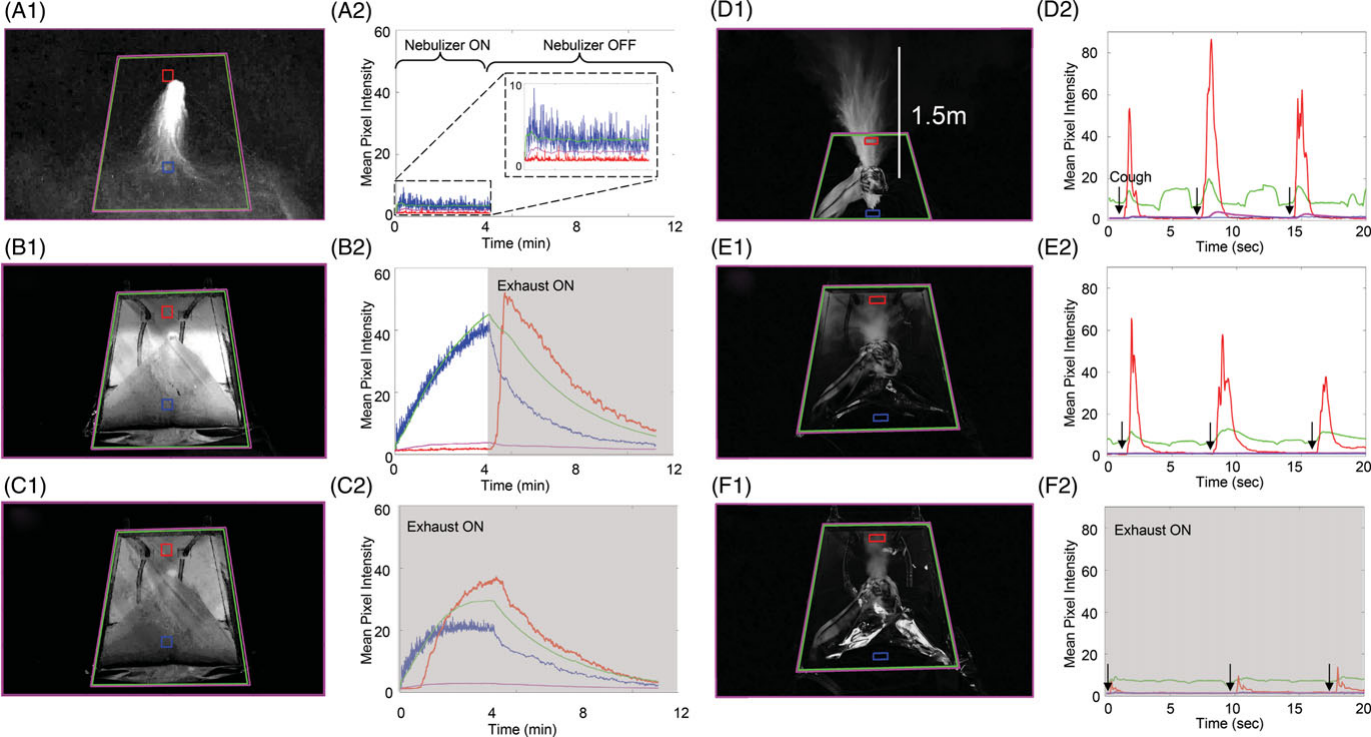
Maximum intensity projections and mean pixel intensities from droplet and aerosol generation experiments. Mean pixel intensity measurements were obtained from 4 regions of interest: inside the vacuum-exhausted isolation locker (VEIL, outlined in green), outside the VEIL (outlined in purple), upper portion of VEIL (outlined in red), lower portion of VEIL (outlined in blue). Nebulization (A–C) continuously produced droplets from 0 to 4 minutes into (A) the ambient air without the VEIL, (B) the VEIL with suction turned on at 4 minutes, and (C) the VEIL with continuous suction. The blue line in B2 rises beyond 40 units, but in C2, it saturates at 20 units. The particle accumulation overall inside the VEIL (green line) plots in C2 is approximately half of B2 and clears more quickly. Vaporized aerosols inhaled and then coughed by a healthy subject (D2–F2, arrows) into (D) ambient air without the VEIL, (E) VEIL without suction, and (F) VEIL with continuous suction shows similar reductions in particle accumulation.

For our experiments, droplet transmission (larger expelled particles that travel short distances) was simulated using a standard oxygen-driven nebulizer. The vaporizer continually produces droplets at a rate of 1 mL per minute, 15 times greater than a typical patient producing 68.3 µL per cough,^8^ conservatively presuming a rapid cough of once every 2 seconds. Aerosol transmission (smaller expelled particles that remain adrift longer and over farther distances due to lower settling velocities) was simulated by a healthy subject inhaling and then coughing out vaporized aerosols while in a supine position. The nebulizer and vaporizer particle size distribution were measured using a laser diffraction particle sizing system (Malvern, Spraytec, UK) and were confirmed to match reported sizes for human respiratory droplets (200 nm–100 µm)^9^ and aerosols (100 nm–1 µm).^10^ We simulated 3 different conditions to assess containment and evacuation of droplets and aerosols: (1) the ambient air, (2) the VEIL without exhaust, and (3) the VEIL with continuous exhaust at 46 L per minute through the viral particle-filtered ports. The experiments were filmed digitally over time at 60 images per second, and the particle concentration at each time point was quantified in selected regions by computing the maximum pixel intensity for each region in each image. Each image is comprised of 2 million grayscale pixels, each numerically defined as a value between 0 (black) and 255 (white). The mean pixel intensity for a region and moment in time is the mean of these pixel values across the selected region of an image. It is correlated with the concentration of particles present and is visible in the 3-dimensional region as a 2-dimensional image at the time it is captured. Mean intensity projections were produced from the sequentially captured images to quantify the droplets and aerosols present within the following regions of interest (Fig. 2): inside the VEIL (green), outside the VEIL (purple), upper VEIL (red), and lower VEIL (blue). The (local) maximum intensity projection is the sum of the maximum pixel intensity values over time for each pixel in the image, producing a convenient visualization of the droplets and aerosols present, but without temporal or depth information perhaps important in more detailed studies.

## Results

### Simulated droplet transmission

Without VEIL containment, droplets continuously exited the nebulizer and descended to and along the bed out of view to the room floor (Fig. 2, A1 and A2). With the VEIL in place, the droplets were confined within the VEIL enclosure (Fig. 2, B1 and B2). The introduction of standard hospital suction reduced the droplet concentration in the VEIL to match the ambient air outside the VEIL (Fig. 2, C1 and C2). The droplet concentration remained unchanged after 4 minutes of droplet nebulization within the VEIL (Fig. 2, C2).

### Simulated aerosol transmission

Vaporized aerosols inhaled and then coughed by a healthy subject are propelled without the VEIL over 1.5 m from the source into ambient air (Fig. 2, D1 and D2). As was the case with droplets, the VEIL contained the aerosols (Fig. E1 and E2) within its enclosure so that no aerosols were evident in the ambient air. Standard hospital suction applied to the VEIL extracted the accumulated aerosols and lowered their concentration to match the ambient air outside the VEIL (Fig. F1 and F2).

## Discussion

In our experiments, the VEIL effectively contained and exhausted exhaled aerosols and droplets using suction through viral particle filters. Continuous evacuation of air from the VEIL reduced the concentrations of particles inside the VEIL. Similar conditions clinically should minimize viral accumulation and additionally avoid CO_2_ rebreathing. For example, suction flow rates meeting US hospital construction code^11^ requirements of 85 L per minute would, with the patient’s upper body inside the VEIL, result in ∼30 enclosure-volume changes per hour.

The VEIL can be rapidly manufactured and deployed to provide safe administration of clinically established noninvasive respiratory support therapies such as NHF, BiPAP, or nebulized medications. The polycarbonate plastic is chemically resistant, easy to disinfect, and reusable. It is compatible with standard hospital beds and changes in bed incline, and it can be used for long-term therapy. Respiratory therapists have applied the VEIL on appropriate COVID-19 patients in our hospital to allow continuous application of NHF (n = 22 at the time of this writing) and BiPAP (n = 4 at this time), with no subsequent contagion of COVID-19 to staff. We speculate that the VEIL can reduce rates of intubation and mortality in critically ill patients with acute hypoxemic respiratory failure secondary to COVID-19 without endangering caregivers. Although this enclosure was developed in light of COVID-19, it is broadly applicable for reducing transmission of other droplet or aerosol-transmitted pathogens.
